# Supplementary figures and images for: Extracellular DJ-1 induces sterile inflammation in the ischemic brain
Source: PLoS Biol. 2021 May 20;19(5):e3000939. doi: 10.1371/journal.pbio.3000939 (PMC8136727; doi:10.1371/journal.pbio.3000939)

*IL-23a* (IL-23p19)

Relative mRNA expression

DJ-1  
0  $\mu$ M

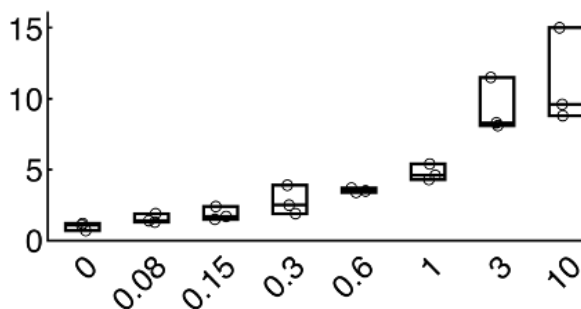

DJ-1  
0.1  $\mu$ M

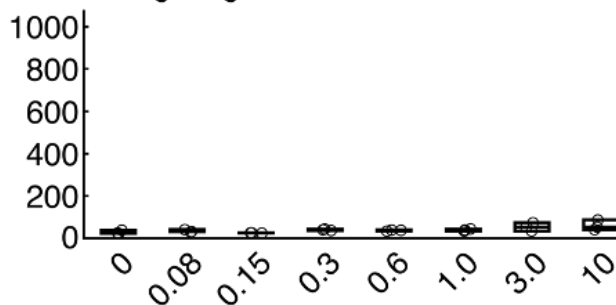

DJ-1  
0.3  $\mu$ M

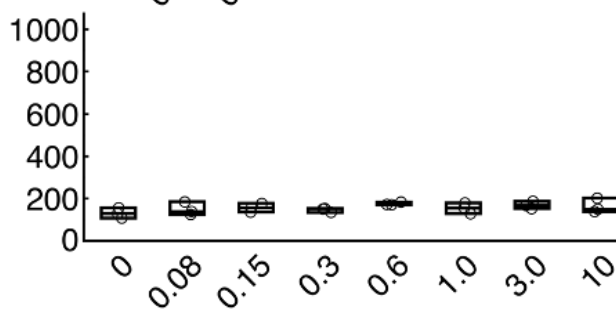

DJ-1  
1.0  $\mu$ M

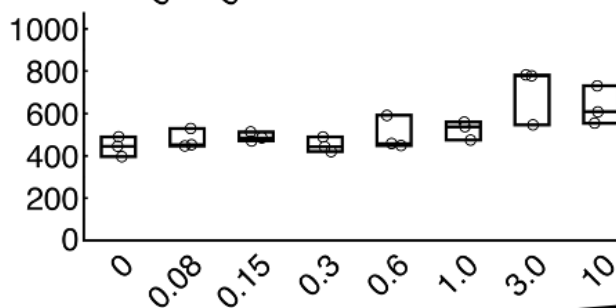

Brain homogenate (mg mL<sup>-1</sup>)

Supplement: S1 Fig — The IL-23p19 mRNA expression of BMDCs treated with brain homogenate alone or costimulated with brain homogenate and 0.1–1.0 μM of recombinant DJ-1 protein. Experiments were performed in triplicate. The data underlying this figure can be found in S1 Data. BMDC, bone marrow–derived dendritic cell. (PDF) [file pbio.3000939.s001.pdf]

Relative luciferase activity

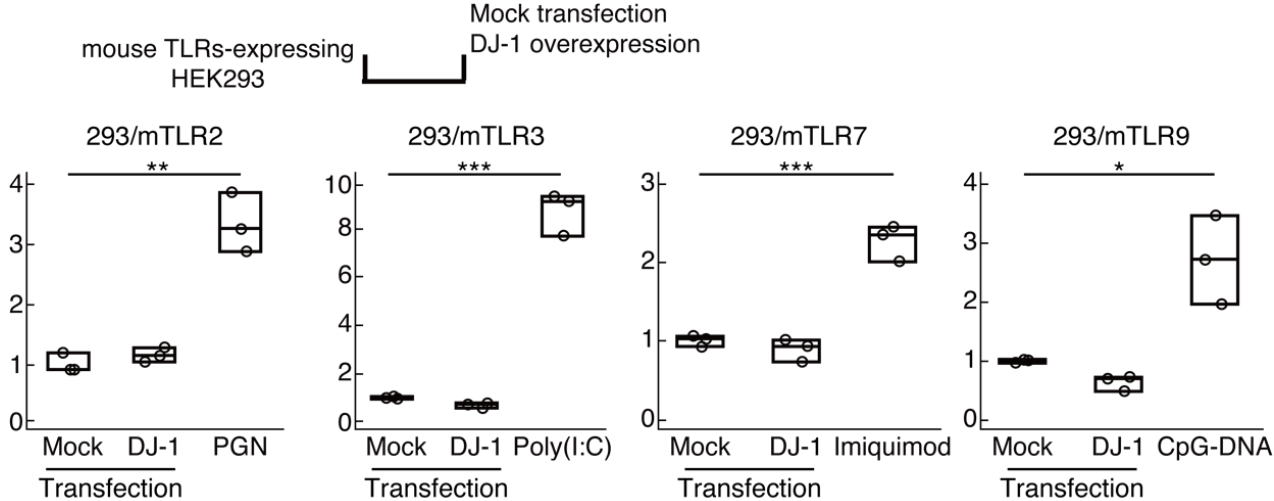

Supplement: S2 Fig — The relative luciferase activity of NF-κB reporter in each transiently murine TLR-expressing HEK293 cell that was transfected with mock or DJ-1 expression vector. TLR ligands were added as a positive control. Experiments were performed in triplicate. The results are representative of 3 independent experiments; one-way ANOVA with Dunnett correction. *p < 0.05, **p < 0.01, ***p < 0.001 vs. mock. The data underlying this figure can be found in S1 Data. ANOVA, analysis of variance; NF-κB, nuclear factor kappa B; TLR, Toll-like receptor. (PDF) [file pbio.3000939.s002.pdf]

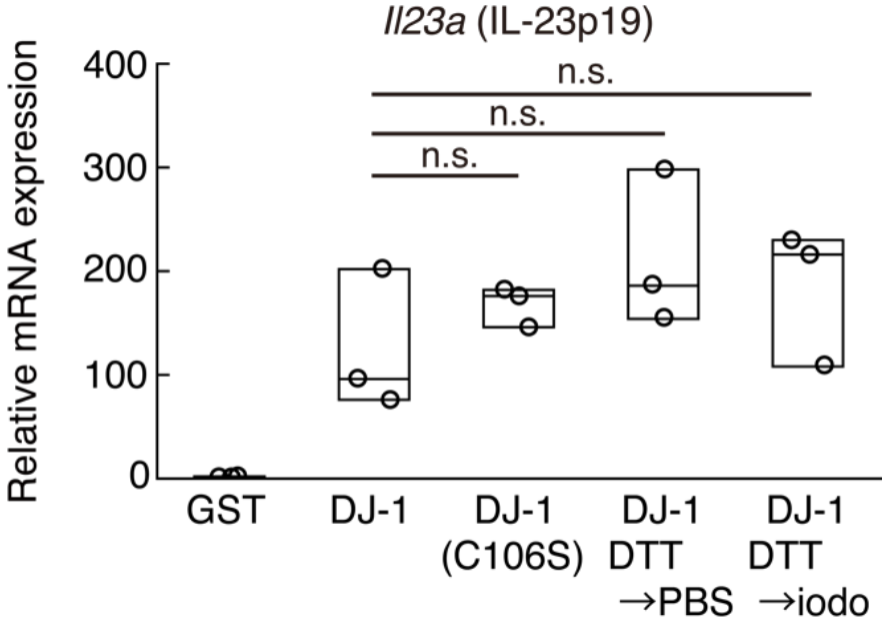

Supplement: S3 Fig — IL-23p19-inducing activities of BMMs treated with DJ-1 protein or mutant DJ-1 protein (C106S) or modified DJ-1 proteins that were treated with PBS or iodo after DTT treatment. Each mRNA expression level is shown relative to that of GST-treated BMMs. Experiments were performed in triplicate. The results are representative of 2 independent experiments; one-way ANOVA with Dunnett correction; n.s., not significant vs. DJ-1. The data underlying this figure can be found in S1 Data. ANOVA, analysis of variance; BMM, bone marrow–derived macrophage; DTT, dithiothreitol; GST, glutathione-S-transferase; iodo, iodoacetamide; PBS, phosphate-buffered saline; (PDF) [file pbio.3000939.s003.pdf]

**A**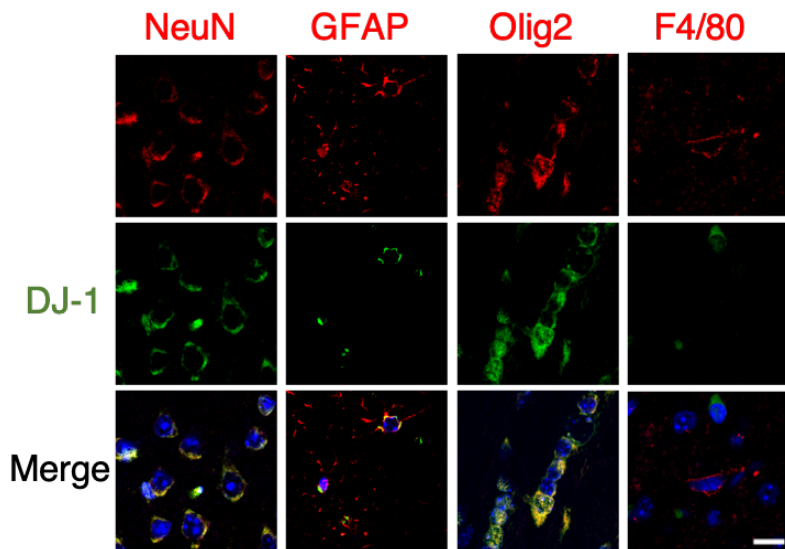**B**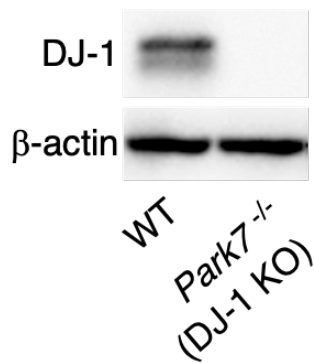**C**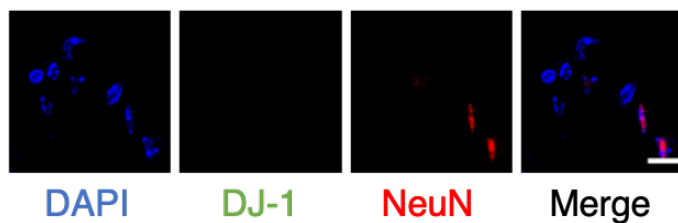

Supplement: S4 Fig — (A) Tyramide-enhanced immunohistochemical staining of DJ-1 in normal brain cells (NeuN for neurons, GFAP for astrocytes, Olig2 for oligodendrocytes, F4/80 for myeloid cells, and DAPI for nuclei). (B) Western blotting analysis of lysates collected from ischemic brain tissues of WT or DJ-1-deficient mice. (C) Immunohistochemical staining of DJ-1, NeuN, and DAPI in the infarct area of DJ-1-deficient mice 24 h after stroke onset. Scale bars: 10 μm [A, C]. GFAP, glial fibrillary acidic protein; WT, wild-type. (PDF) [file pbio.3000939.s004.pdf]

**A**

Infarct area (permanent MCAO)

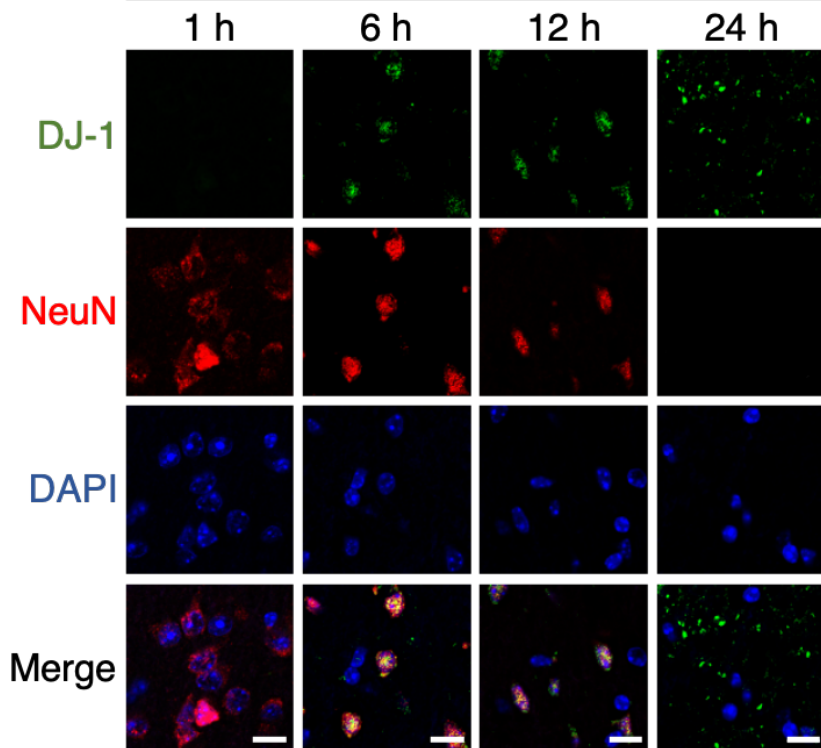**B**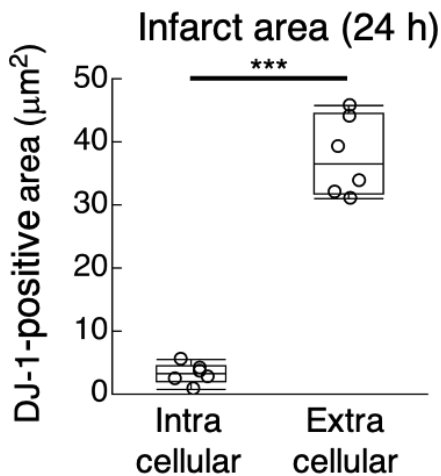

Supplement: S5 Fig — (A) Immunohistochemical staining of DJ-1, NeuN, and DAPI in the infarct area of the permanent MCAO model. Time-dependent changes in the ischemic brain were investigated at the indicated time points. Scale bar: 10 μm. (B) Quantification of intracellular or extracellular DJ-1-positive areas in the infarct area 24 h after stroke onset. n = 6 mice for each group. Two-sided Student t test; ***p < 0.001 vs. intracellular DJ-1-positive area. The data underlying this figure can be found in S1 Data. MCAO, middle cerebral artery occlusion. (PDF) [file pbio.3000939.s005.pdf]

**A**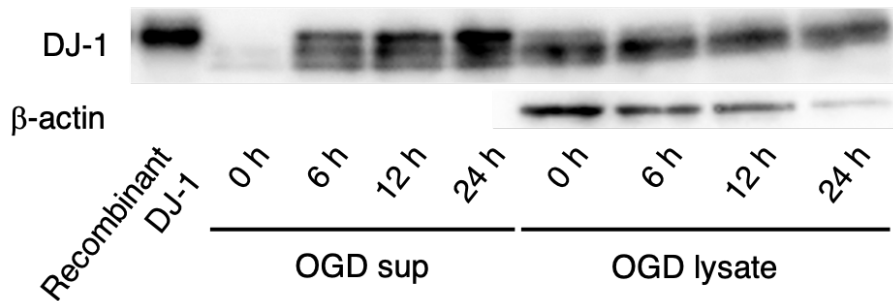**B**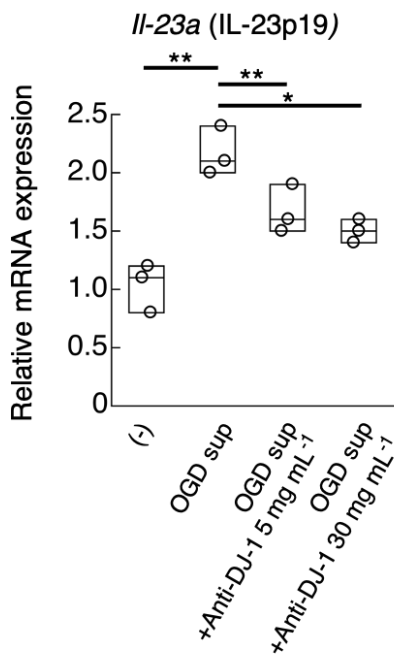

Supplement: S6 Fig — (A) Western blotting analysis of the culture supernatant (OGD sup) and cellular lysate (OGD lysate) of primary neurons subjected to the OGD. The samples were collected from 1 dish of cultured primary neurons at each time point after OGD and same liquid volumes of each sample were analyzed by western blotting. (B) The IL-23p19 mRNA expression of BMDCs treated with the culture supernatant of primary neurons 24 h after OGD with/without anti-DJ-1 antibody. One-way ANOVA with Dunnett correction; *p < 0.05, **p < 0.01 vs. BMDCs treated with the cell supernatant of primary neurons after OGD. Experiments were performed in triplicate. The results are representative of 3 independent experiments. The data underlying this figure can be found in S1 Data. ANOVA, analysis of variance; BMDC, bone marrow–derived dendritic cell; OGD, oxygen-glucose deprivation. (PDF) [file pbio.3000939.s006.pdf]

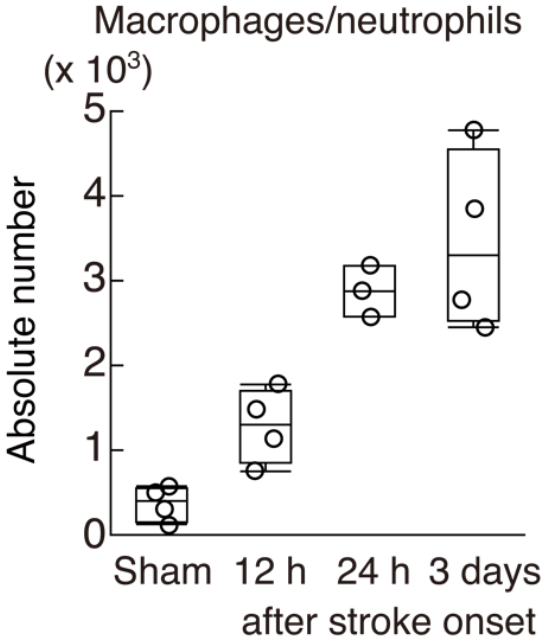

Supplement: S7 Fig — The absolute number of CD45highCD11bhigh population (macrophages/neutrophils) collected from the ischemic brain at each time point after the onset of ischemic stroke (n = 4 mice for sham, 12 h, and 3 days; n = 3 mice for 24 h). The data underlying this figure can be found in S1 Data. (PDF) [file pbio.3000939.s007.pdf]

**A**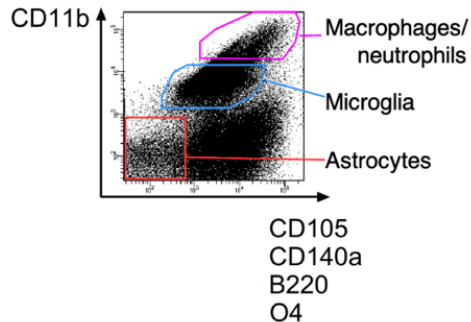**B**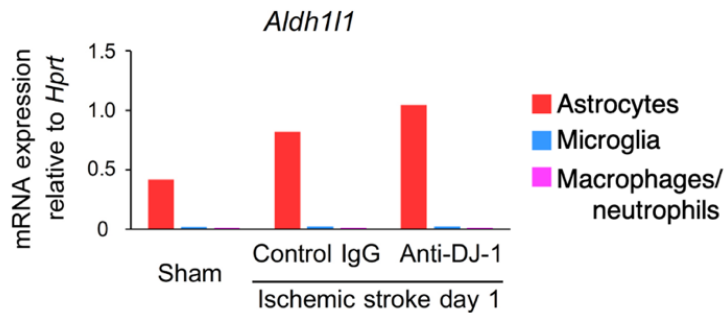**C**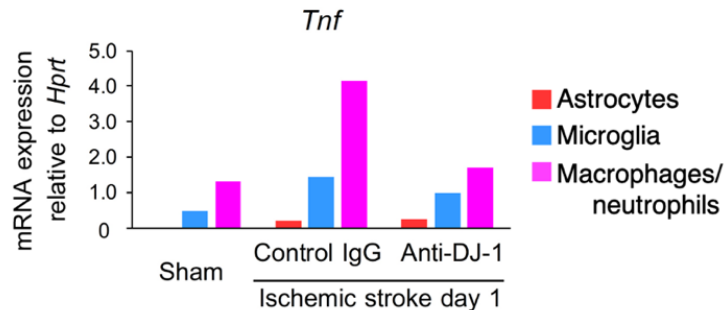

Supplement: S8 Fig — (A) The gating strategies for collecting macrophages/neutrophils (CD11b-high), microglia (CD11b-intermediate), and astrocytes (CD11b−, CD105−, CD140a−, B220−, O4−) by FACS. This method for sorting astrocytes was described elsewhere [34,35]. (B) The successful isolation of astrocytes was confirmed by the mRNA expression levels of Aldh1l1 (an astrocyte marker) in the astrocytes, microglia, and macrophages/neutrophils collected from sham-operated mice or day 1 post-ischemic brains of mice treated with DJ-1-specific antibody or control IgG antibody immediately after stroke onset. (C) The mRNA expression levels of TNFα in the pooled astrocytes, microglia, and macrophages/neutrophils population isolated by FACS from 3 sham-operated mice or day 1 post-ischemic brains of 3 mice treated with control IgG antibody or DJ-1-specific antibody immediately after stroke onset. The data underlying this figure can be found in S1 Data. FACS, fluorescence-activated cell sorting; IgG, immunoglobulin G; TNFα, tumor necrosis factor alpha. (PDF) [file pbio.3000939.s008.pdf]

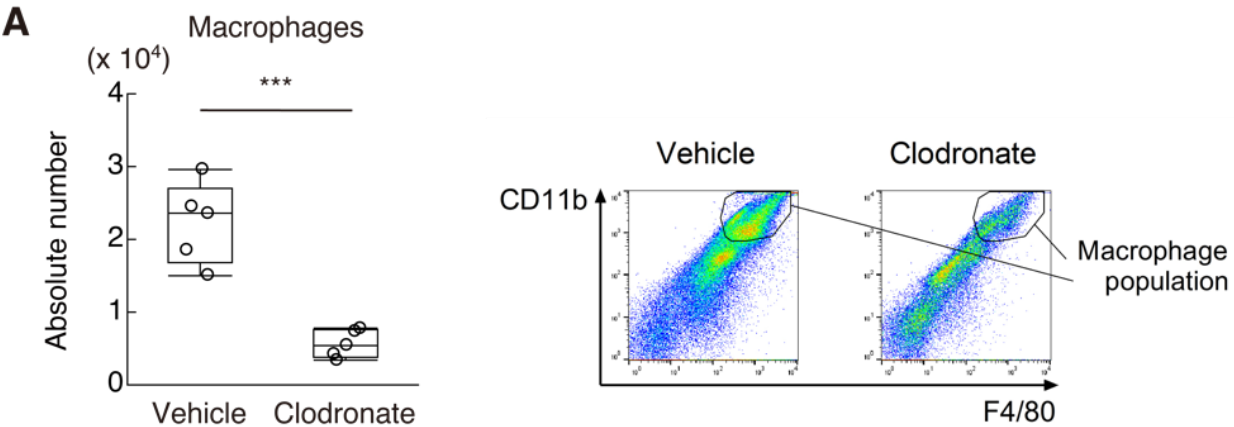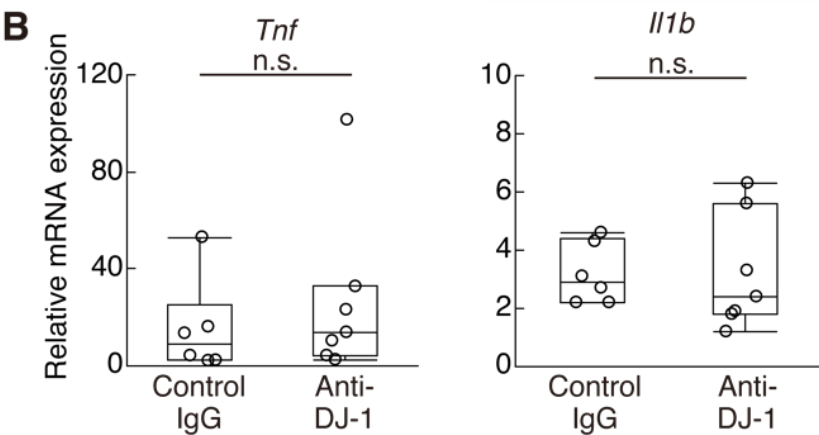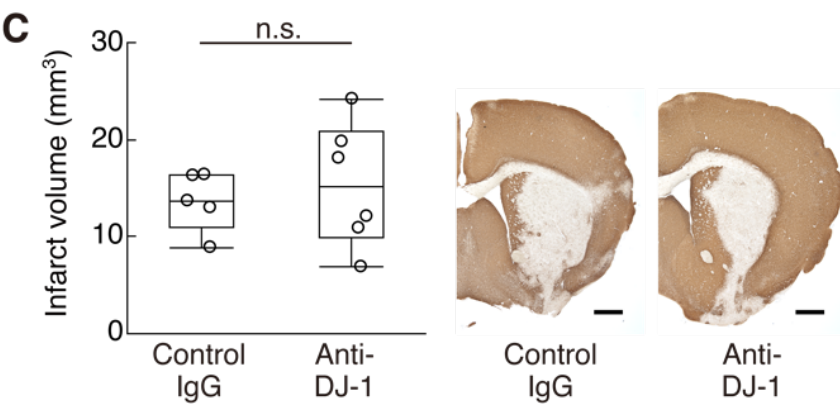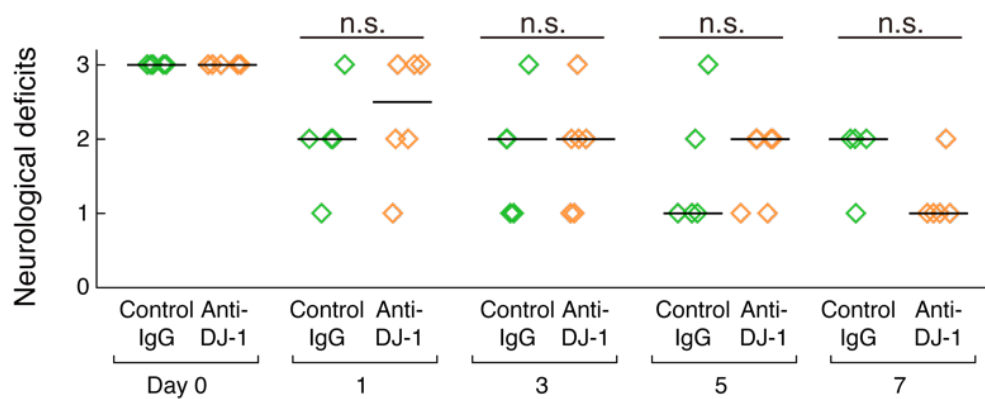

Supplement: S9 Fig — (A) The absolute number of infiltrating macrophages (CD11bhighF4/80+) collected from the day 3 post-ischemic brain of mice treated with vehicle or clodronate liposome (n = 5 mice for each group). ***p < 0.001 vs. vehicle-treated mice. The right panel shows the representative result of FACS. (B) The relative mRNA expression levels in the day 3 post-ischemic brain tissue of mice treated with clodronate liposome and indicated antibody (n = 6 mice for control IgG, n = 7 mice for anti-DJ-1 antibody), compared to sham-operated mice. n.s., not significant vs. control IgG. (C) Infarct volume on day 7 after stroke onset and neurological deficits of mice treated with clodronate liposome and indicated antibody. n.s., not significant vs. control IgG (n = 5 mice for control IgG, n = 6 mice for anti-DJ-1 antibody). Two-sided Student t test [A–C: infarct volume]. Wilcoxon rank sum test with Bonferroni correction [C: neurological deficits]. The data underlying this figure can be found in S1 Data. FACS, fluorescence-activated cell sorting; IgG, immunoglobulin G. (PDF) [file pbio.3000939.s009.pdf]

**A**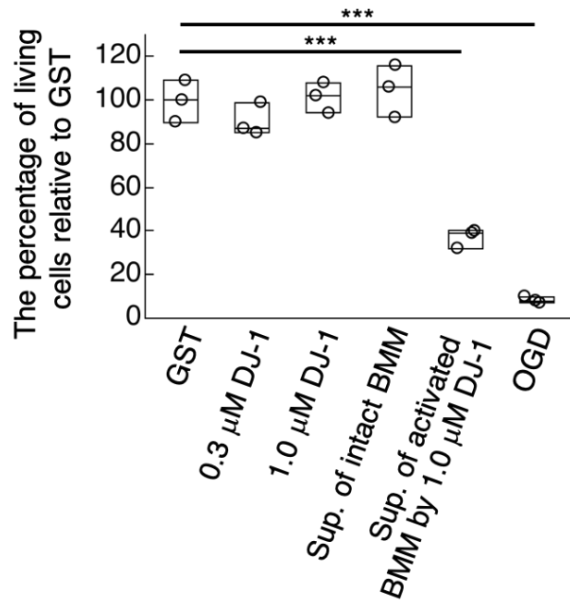**B**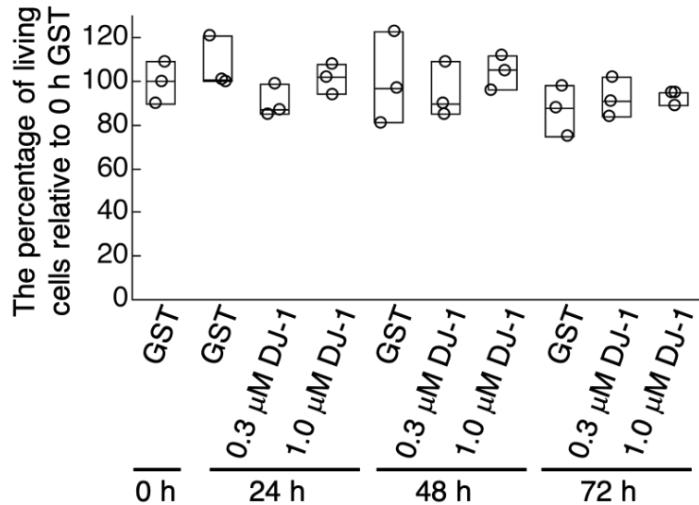

Supplement: S10 Fig — (A) The percentage of living primarily cultured neuronal cells that were treated for 24 h with recombinant GST or DJ-1 or culture supernatant from intact BMMs or BMMs treated with DJ-1 for 3 h. OGD was performed as a positive control of neuronal cell death (one-way ANOVA with Dunnett correction). ***p < 0.001 vs. GST-treated cells. (B) The percentage of living primarily cultured neuronal cells that were treated with recombinant GST or DJ-1 at each time point. Experiments were performed in triplicate [A, B]. The data underlying this figure can be found in S1 Data. ANOVA, analysis of variance; BMM, bone marrow–derived macrophage; GST, glutathione-S-transferase; OGD, oxygen-glucose deprivation. (PDF) [file pbio.3000939.s010.pdf]

Fig 3A

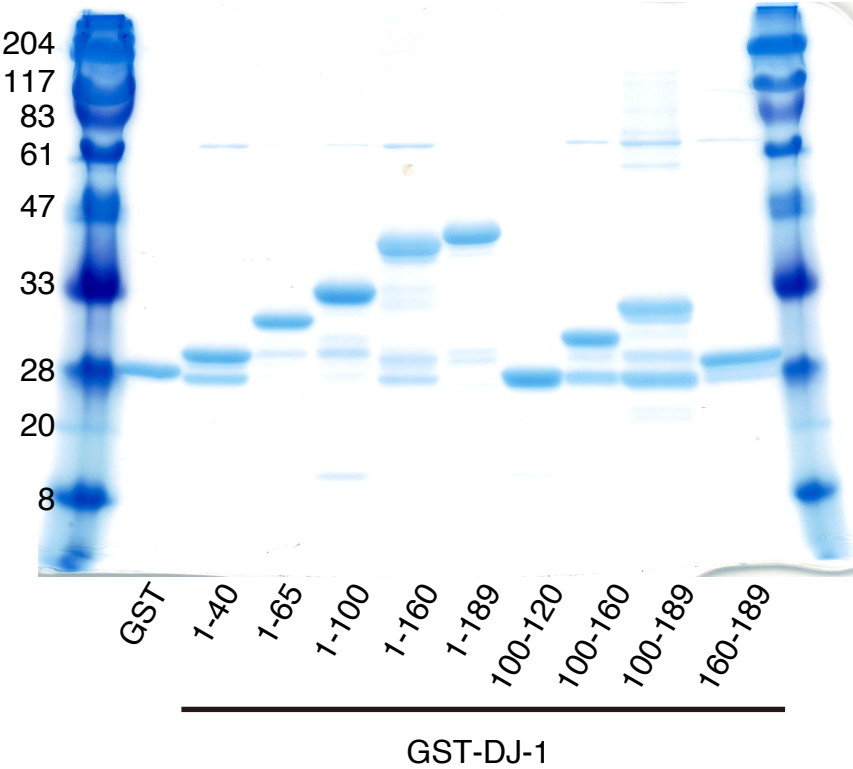

S4B Fig

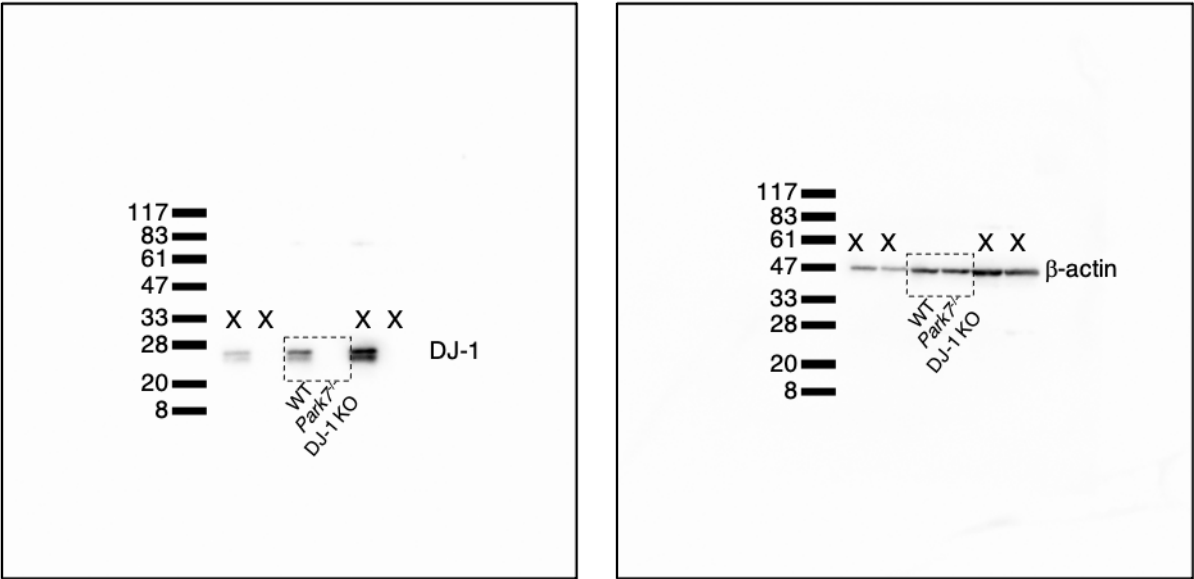

S6A Fig

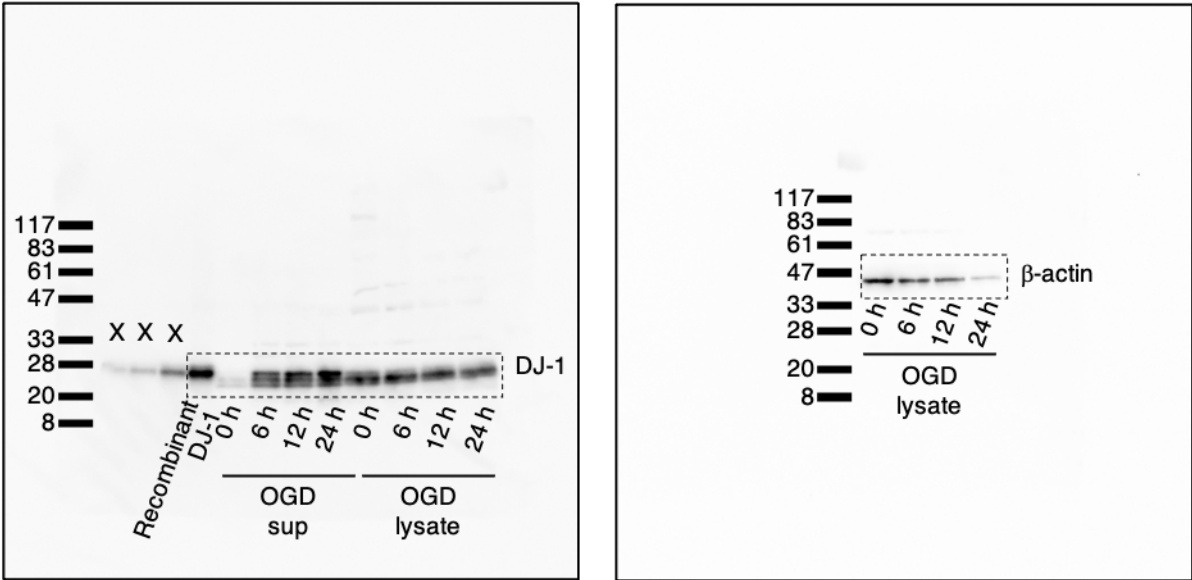

Supplement: S1 Raw Images — Unedited, original blots images for Figs 3A, S4B and S6A. (PDF) [file pbio.3000939.s014.pdf]
