# Supplementary material for: Extracellular DJ-1 induces sterile inflammation in the ischemic brain
Source: PLoS Biol. 2021 May 20;19(5):e3000939. doi: 10.1371/journal.pbio.3000939 (PMC8136727; doi:10.1371/journal.pbio.3000939)
Supplement: S1 Table — (A) Kaplan–Meier survival curve for WT vs. DJ-1-deficient mice and the percent of CBF reduction after CCA and MCA occlusion that was used in Fig 5. (B) Kaplan–Meier survival curve for control IgG antibody–administered mice vs. anti-DJ-1-administered mice and the percent of CBF reduction after CCA and MCA occlusion that was used in Fig 6. Values are shown as the mean ± standard error of the mean [A, B]. The data underlying this figure can be found in S1 Data. (PDF) [file pbio.3000939.s011.pdf]

S1 Table.

A

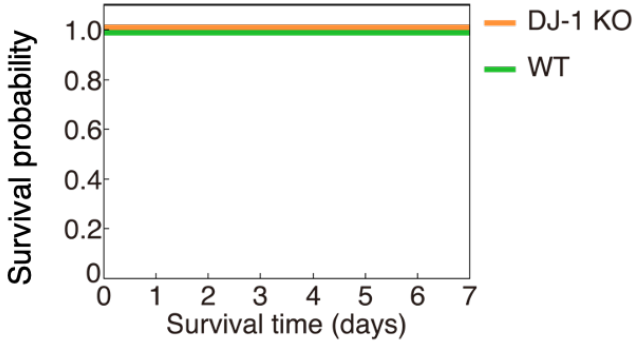

CBF reduction (%)	N	before CCA occlusion	after CCA occlusion	after MCA occlusion
WT	6	100	84.1 ± 2.5	32.0 ± 0.9
DJ-1 KO	6	100	74.2 ± 4.8	33.0 ± 1.5

B

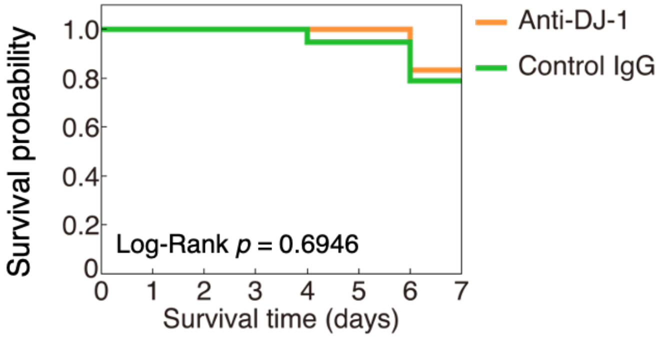

CBF reduction (%)	N	before CCA occlusion	after CCA occlusion	after MCA occlusion
Control IgG	19	100	84.1 ± 1.4	31.8 ± 0.8
Anti-DJ-1	18	100	86.7 ± 1.3	28.1 ± 1.2
