# Supplementary material for: Extracellular DJ-1 induces sterile inflammation in the ischemic brain
Source: PLoS Biol. 2021 May 20;19(5):e3000939. doi: 10.1371/journal.pbio.3000939 (PMC8136727; doi:10.1371/journal.pbio.3000939)
Supplement: S2 Table — List of primers used for quantitative PCR. (PDF) [file pbio.3000939.s012.pdf]

**S2 Table.** List of primers used for quantitative PCR.

| Genes | Forward primer | Reverse primer |
|---|---|---|
| *Tnf* | CATCTTCTCAAAATTCGAGTGACAA | TGGGAGTAGACAAGGTACAACCC |
| *Il1b* | CAGGCAGGCAGTATCACTCA | AGGCCACAGGTATTTTGTCG |
| *Il23a* | AGC GGG ACA TAT GAA TCT ACT AAG AGA | GTC CTA GTA GGG AGG TGT GAA GTT G |
| *Il12b* | GACCATCACTGTCAAAGAGTTTCTAGAT | AGGAAAGTCTTGTTTTTGAAATTTTTTAA |
| *Hprt1* | TGAAGAGCTACTGTAATGATCAGTC | AGCAAGCTTGCAACCTTAACCA |
