# Supplementary material for: Extracellular DJ-1 induces sterile inflammation in the ischemic brain
Source: PLoS Biol. 2021 May 20;19(5):e3000939. doi: 10.1371/journal.pbio.3000939 (PMC8136727; doi:10.1371/journal.pbio.3000939)
Supplement: Attachment — Submitted filename: R2R.pdf [file pbio.3000939.s015.pdf]

Point by point reply to the reviewer's comments

Reviewer #1 (Evidence, reproducibility and clarity (Required)):

The authors aimed in this study to establish the role of DJ-1 as a DAMP in ischemic stroke. This is a follow-up story on their previous work characterizing DAMPs in different fractions of brain homogenates after ischemic stroke. They show that recombinant DJ-1 can activate BMDMs and its neutralization in vitro reduces the neuroinflammatory response to stroke.

There is already a considerable body of literature on the pathophysiological function of DJ-1 in ischemic stroke models. The novelty of this study however is derived from proposing a pro-inflammatory function for DJ-1 as a DAMP in contrast to the main concept in the field of DJ-1 as an antioxidative peptide.

MAJOR:

- The actual function of DJ-1 as a DAMP of its own is not clearly demonstrated in the present work. The in vitro part of this study uses recombinant DJ-1 (i.e. the purified peptide) in either BMDM or HEK cell cultures. However, it is very likely that in vivo DJ-1 forms complexes with other DAMP molecules (nucleic acids, lipids, histones, S100 proteins, etc.) as is known very most other DAMP classes. Therefore, interpretation of the in vitro experiments for the in vivo situation in this case is not quite possible. I suggest to add an in vitro experiment in which DJ-1 is added to BMDMs either together with brain homogenate or the 15-25 kDa fraction, at increasing doses in order to terst the effect of DJ-1 in a comparable matrix as for the in vivo situation.

<u>Response</u>: As suggested by reviewer#1, we have investigated whether the addition of brain homogenate with a recombinant DJ-1 protein increases DAMP activity in BMDMs to exclude the possibility that DJ-1 is conjugated with other DAMP molecules (nucleic acids, lipids, histones, S100 proteins, etc). As shown right, the addition of supernatant of brain homogenate with recombinant DJ-1 protein did not show remarkable changes in the *Il23a*-inducing activity compared to

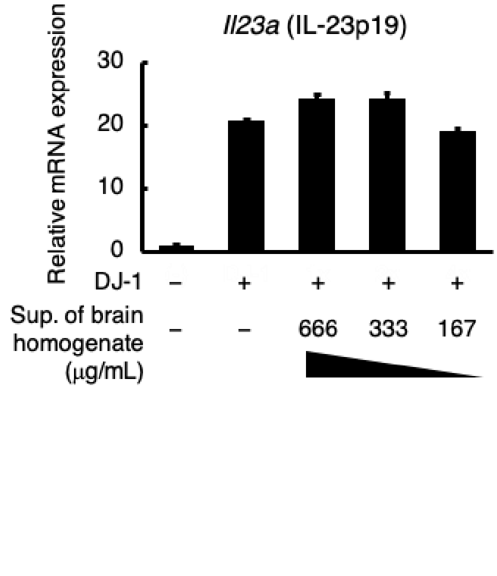

the addition of recombinant DJ-1 protein only. We have also performed LS/MS analysis; however, no nucleic acids and proteins could be detected in our recombinant DJ-1 protein which had a remarkable DAMP activity. Thus, it is unlikely that DJ-1 forms complexes with other DAMP molecules.

In our article, we have already demonstrated unequivocally that DJ-1 plays a major role in triggering the inflammation after ischemic stroke by using DJ-1-deficient mice and anti-DJ-1 neutralizing antibody. Especially, DJ-1-deficiency remarkably decreases the inflammatory cytokine expression in the infiltrating cells collected from the ischemic brain, indicating an indispensable role of DJ-1 in triggering the cerebral post-ischemic inflammation. This evidences support our conclusion that DJ-1 is the key DAMP molecule that triggers cerebral post-ischemic inflammation.

- The role of DJ-1 as a DAMP would require its (very) early release and broad interaction with immunocompetent cells in the ischemic brain. However, kinetics for extracellular DJ-1 expression after stroke is rather slow (Figure 4) where infiltration of innate immune cells in fact precedes DJ-1 expression.

Response: We appreciate this reviewer's comment; however, we are afraid that reviewer#1 misunderstands the time-dependent changes of the infiltration of immune cells. The extracellular release of DJ-1 was remarkable 24 hours after stroke onset, which is coincided with the beginning of macrophage infiltration. We have previously investigated the time-dependent changes of macrophage infiltration and found that the number of infiltrating macrophages reached its peak on day 3 and thereafter decreased (shown right: cited from Shichita *et al*. *Nature Medicine* 2009). Thus, the extracellular release of DJ-1 in the ischemic brain is coincided with the infiltration of immune cells, indicating the important role of extracellularly released DJ-1 in stimulating immune cells directly.

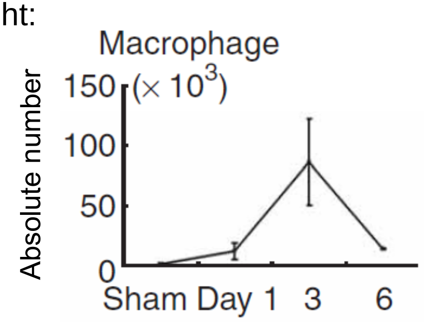

Moreover, extracellular DJ-1 expression seems to be confined to the ischemic core. Thereby, interaction of DJ-1 with immune cells in the peri-ischemic area

-- the vulnerable area at risk after stroke and major site of secondary neuroinflammation -- is not plausible.

<u>Response</u>: We appreciate the reviewer's comment. Extracellular release of DJ-1 was observed around the peri-infarct area (**Figure 4B**), although the reviewer did not notice that the DJ-1-positive area was found in the peri-infarct area. There are many surviving neurons in the peri-infarct area; therefore, DJ-1 was majorly detected within neurons. In the necrotic region around the peri-infarct area (shown as "infarct" in **Figure 4B**), DJ-1 was apparently observed in the extracellular space. Thus, DJ-1 can trigger post-ischemic inflammation around the peri-infarct area. To make this easy to understand, we will insert a schematic indicating the peri-infarct area and infarct area in the cerebral cortex of our brain ischemia model.

- infarct volumes for DJ-1 KO mice and littermate controls need to be reported, ideally also behavior analysis between these groups should be added

<u>Response</u>: As the reviewer suggested, we will show the results of the infarct volume and neurological deficits in DJ-1 KO mice as below.

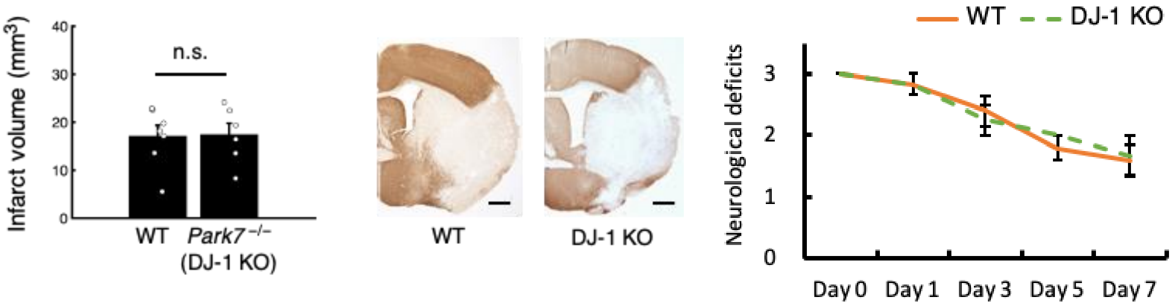

MINOR:

- the results of the behavior analysis are not convincing. It seems that both groups have similar deficits in the acute phase as well as up to 5 days after the lesion, significant difference only at 7d post-stroke. This does not fit with a function of DJ-1 as a DAMP in the acute phase driving an acute inflammatory response.

Response: We have detected the tendency of improved neurological deficits on day 3 after stroke onset in anti-DJ-1-antibody-administered mice. We will clarify whether this is statistically significant by further experiments. Since DJ-1 not only triggers the inflammation by immune cells but also promotes the inflammation in ischemic brain tissue on day 3 after stroke onset (**Figure 6A,B**), the DAMP activity of extracellularly released DJ-1 coincides with the worsening of neurological deficits after ischemic stroke.

- results in Figure 4 E,F need to be quantified to show relevant interaction of innate immune cells with DJ-1 expressing cells. This should be demonstrated not only for the infarct core (which is anyways already dead tissue) but also for the perilesional tissue at risk.

Response: We appreciate the reviewer's comment. We will quantify this in our revised manuscript.

Reviewer #1 (Significance (Required)):

The significance of this work is still unclear at this stage because the claim of novelty and impact comes from the hypothesis that DJ-1 acts as a DAMP. However, this is neither unequivocally demonstrated in this study and some data sets (e.g. kinetics of stroke outcome, no effect in DJ-1 knockouts) even contradict this hypothesis.

Reviewer #2 (Evidence, reproducibility and clarity (Required)):

The present manuscript by Nakamura et al. identified DJ-1 or PARK7 as a mediator of sterile inflammation after brain ischemia. The authors statement that DJ-1 functions as a novel danger associated molecular pattern that boosts sterile inflammation in bone marrow derived macrophages via activation of the pattern recognition receptors TLR2 and 4. The authors started exploring the inflammatory action of DJ-1 recombinant protein using isolated bone marrow derived macrophages treated with GST-tagged DJ-1

and mRNA changes as a response to the concentration or to the time after stimulation for various inflammatory markers. ELISA (TNFalpha and IL-23) investigated two of these on the protein level. In the next set of experiments, the authors aimed identifying the receptor that is targeted by DJ-1 recombinant protein: Human embryonic kidney cells were stably transfected with murine TLR2 and reporting for nuclear factor kappa B using promoter luciferase activity or hTLR4/MD2-CD14 stably expressing 293 cells with NF-kB reporter. Both were not stimulated by GST-control protein but by DJ-1 and the positive controls, respectively (PGN or LPS). In contrast, DJ-1 did not stimulate NF-kB if stably transfected for other TLRs (mTLR3, 7, 9) while the respective positive controls suffice. Double Tlr2/4 BMM did not display increased levels of Tnf or Il1b, Il23a or Il12b mRNA upon stimulation with DJ-1 while data on the protein level is not shown by the authors. In Figure 3, DJ-1 regions putatively essential for its stimulatory action were identified using peptide mapping and deletion mutants with Il23a mRNA as a readout in BMMs. In Figure 4, mice after MCAo were subjected to histological analysis of DJ-1's localization. DJ-1 deficient mice were used to show that mRNA expression of key inflammatory signalling molecules are decreased. Finally, mice were treated with a custom-made DJ-1 antibody and transcription of mRNA and infarct volume and TUNEL-positive cells were investigated.

In summary, this is a novel, interesting and important finding that DJ-1 has other non-cell-autonomous functions in the context of stroke. The statement that it induces sterile inflammation is supported by several in vitro and in vivo experiments. In the opinion of the reviewer, some of these experiments need further controls and display of data that is currently not shown to increase the level of transparency. In general, it is highly recommended to follow the ARRIVE guidelines to avoid bias in the results. Some conclusions cannot be derived from the set of data presented and should be re-designed or complemented as outlined below.

Major points:

1. Figure 2 D: What is the response of single knockout BMMs of Tlr2 or Tlr4 on DJ-1?

Response: We have investigated the mRNA expression levels of *Il23a* in *Tlr2*- or *Tlr4*-deficient BMMs stimulated by DJ-1, and found that these expression levels were partially decreased by either Tlr2- or Tlr4-deficiency as shown below. We will also investigate other inflammatory cytokines in our revised manuscript.

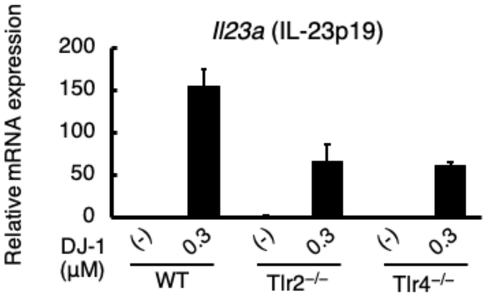

2. Why did the authors skipped the ELISAs of stimulated Tlr2/4 double knockout BMMs?

Response: We will investigate the expression levels of inflammatory cytokines in the culture supernatant of *Tlr2/4*-deficient BMMs by ELISA.

3. The display of the data is not satisfactory. For the sake of transparency, bar graphs should not be used. Instead, scatter dot plots like in Figure 4-6 and 95% confidence intervals, SDs or box with whiskers better illustrate the amount of independent samples. It is also not transparent what n means in the context of the displayed data (e.g. for Figure 2D). If these are independent experiments it is not enough to show technical replicates or repeated plating as independent n but rather independent experiments (isolation of BMMs from independent animals). The authors should clarify this, nested data from one animal should be avoided, or explicitly stated (and needs different statistics, too).

Response: We appreciate this reviewer's comment. We will improve the figures by using scatter dot plots or box with whiskers. The number of independent experiments will be also described in figure legends of our revised manuscript.

4. If DJ-1 is released from isolated neurons it should be enriched from the

medium of primary neurons subjected to oxygen-glucose deprivation and if transferred to BMMs act on the secretion of cytokines. This action might be blocked if neurons from DJ-1 knockout mice have been used or if supernatants have been incubated with anti-DJ-1 antibody.

Response: We agree with the reviewer's comment. A quite large number of neuronal cells are dead in the ischemic brain; however, the number of primary cultured neurons may be too small to activate BMMs even if the extracellular release of DJ-1 is induced by oxygen-glucose deprivation (OGD) in cultured neurons. We are now examining OGD in primary neurons to clarify this question. If the activation of BMMs is successfully observed, we will investigate whether the depletion or neutralization of DJ-1 attenuates the activation of BMMs.

5. Why did the authors conclude that the immune-stimulatory action of released DJ-1 does not stimulate resident immune cells of the brain like astrocytes or microglia?

Response: We are investigating the expression levels of inflammatory cytokines in astrocytes and microglia to clarify this question.

6. Figure 4 C: How do the authors exclude that DJ-1 is released in vesicles? Experiments that really explore whether it's extracellular DJ-1 are not presented. Please complement the experiments with live cell imaging of DJ-1 fusion to a fluorophore expressed in neurons derived from DJ-1 knockout animals and fluorescence fusion of either TLR2 or 4 in BMMs to show that released DJ-1 from neurons is binding to these receptors by FLIM/FRET or by proximity ligation assays.

Response: As this reviewer suggested, we are investigating the direct interaction between DJ-1 and TLR by proximity ligation assay.

7. Figure 5: Did the authors isolate the same amount of cells from sham mice for comparison of mRNA levels? What is the difference in stroke volume in

WT and DJ-1 knockout mice given the cell-autonomous action of DJ-1 in neurons etc.? Data not shown does not suffice because it is an important experiment. What happens if the authors compare the effects to the contralateral hemisphere of individual mice and does the enrichment strategy really underpins the statement that these are invading immune cells? Were the infarct sizes controlled to exclude a bias towards decreased stroke volumes as inflammation is a dependent variable on stroke volume? Why do the authors show relative levels to sham and not the raw data of all animals?

Response: We appreciate the comment of this reviewer. We will show the results of the comparison of infarct volumes between DJ-1 KO and WT mice (there was no significant difference, shown below). We have compared the expression levels of inflammatory cytokines by raw data of all animals (shown below: mRNA expression level of inflammatory cytokines relative to *Hprt*) or with one in the cells collected from contralateral hemisphere; nevertheless, the suppression of inflammatory cytokine levels are detected, revealing that DJ-1-deficiency significantly attenuated the cerebral post-ischemic inflammation even if infarct sizes were similar between DJ-1 KO and WT mice.

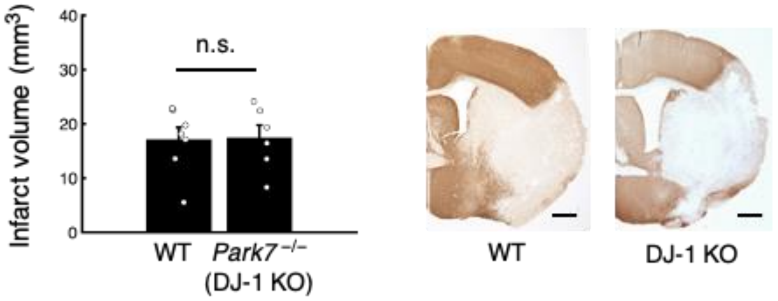

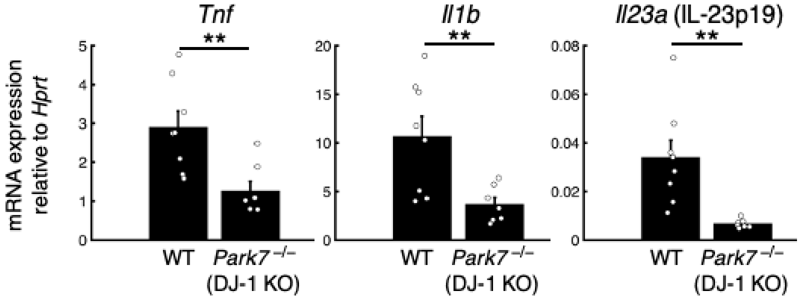

8. The problem of causal inference is true for the treatment with DJ-1 antibody. It is possible that DJ-1 antibody treatment results in smaller infarcts for different reasons than those that are hypothesized by the authors? Thus, inflammation is reduced and for example Il23a or Tnf are reduced as a consequence of smaller infarction. What happens, if TLR2/4 knockout mice subjected to antibody treatment or if the infarcts are titrated by timing of the occlusion period to the same size? Is Anti-DJ-1 antibody still decreasing Tnf on the mRNA level? Of note, clodronate experiments should be visualized to allow readers to understand how successful macrophages vanished from the brain.

Response: As the reviewer suggested, we are investigating the infarct volume of TLR2/4-deficient mice which are treated with antibodies. We have also confirmed the successful depletion of macrophages by the administration of chlodronate.

9. The authors state that inflammation rather than extracellular DJ-1 triggers neuronal cell death (Fig S6). First, Neuro2A cannot be considered a neuron and it is extensively demonstrated that neurons are distinct to immortalized cells and rapidly lose their phenotype. This experiment would be more meaningful if primary neurons would have been investigated and studied over a longer period than 24h, which is too short to exclude that DJ-1 induces apoptosis over several days.

Response: We appreciate the reviewer's comment. We are investigating the effect of culture supernatant of DJ-1-stimulated BMMs in primary neurons.

10. Figure S7: What is the effect of clodronate on the immune cells and what is the effect of combined treatment on the stroke volume?

Response: We have also confirmed the successful depletion of macrophages by the administration of chlodronate, and we are clarifying the effect of chlodronate combined with antibody treatment in infarct volume.

11. Table S2: The authors show the mortality without statistics and percentage although group sizes were not equal in the beginning. A statistical analysis is not shown although the data were interpreted. Please change this. Reporting should be in line with the ARRIVE guidelines and mortality, exclusion and inclusion criteria, time and reason of death etc. should be reported for each in vivo experiment.

Response: This information will be described in our revised manuscript.

12. A Score should be displayed as median and not as mean since these are not metric data. Post-hoc correction for multiple testing should be performed. The authors overstretch this functional readout a lot. It is not described whether blinding or randomization was performed and easy to apply behavioural tests were not used. The reviewer understands that this might be not the scope of this publication but one cannot draw the conclusion that there is functional improvement by the treatment.

Response: We apologize for our mistake. The statistical processing methods will be improved as the reviewer suggested in our revised manuscript.

13. Did the authors investigate whether DJ-1 antibody is not resulting in a western blot immunoreactivity in brain tissue derived from knockout mice? Did they test immunohistochemistry with their DJ-1 antibody?

Response: We have confirmed the specificity of the DJ-1 antibody by using DJ-1-deficient tissue.

Minor points:

Display of graphs as scatter dot plots and SD or 95% CI would be preferred.
BMM - please spell out at first usage on page 4 in the heading
PGN please spell out at first usage (TLR2 ligand)

Response: We will correct our manuscript as this reviewer suggested.

Reviewer #2 (Significance (Required)):

Non-cell autonomous action of extracellular DJ-1 on TLR2/4 in the context of stroke is not known and is important not only for scientists interested in brain ischemia but in a broader context of neuroinflammation. As a scientist active in research focussing on experimental stroke models some important in vitro experiments with primary neuronal cultures are missing and cannot be derived from a neuronal cell line. It is also not clear, what is the effect of blocking action on functional readouts using carefully selected behavioural tests. Since all these tests and the impact of blocking extracellular DJ-1 have a high significance, the custom-made antibody should be characterized more carefully as outlined above.

Reviewer #3 (Evidence, reproducibility and clarity (Required)):

Summary

The paper by Nakamura and colleagues investigates the role of DJ1 in activating peripheral immune cells in ischemia. They carry out a series of comprehensive experiments demonstrating that specific parts of the DJ1 protein are capable of activating monocytes, and that this activation is detrimental in ischemic stroke. Overall the paper is well written and well executed. I have some queries the reviewers may like to consider and respond to, these are detailed below.

Major comments:

1. Details of the percoll gradient for immune cells in the brain are not clear. How are the authors guaranteeing they have infiltrating monocytes vs microglia/astrocytes/etc.

<u>Response</u>: We apologize for our mistake. We have confirmed the enrichment of infiltrating immune cells (monocytes and microglia) by Percoll-gradient centrifugation, which was described in our previous articles (Shichita *et al*. *Nature*

Medicine 2009, 2012). Although these cells also include brain cells such as astrocytes, we also confirmed that the major source of inflammatory cytokines was infiltrating monocytes or microglia but not other brain cells. In our revised manuscript, we will explain this and replace “infiltrating immune cells” with “immune-cell-enriched cellular population collected from the ischemic brain”.

2. There is a comment in the discussion saying that data is not shown on infarct volumes in DJ1 knockout mice, I believe these would considerably add to the manuscript and I believe they should be included.

Response: We appreciate the reviewer’s comment. We will show the result of the infarct volume of DJ-1 KO mice in our revised manuscript.

3. The immunohistochemistry for DJ1 is supposed to be 'infarct', it would be useful to have a schematic to demonstrate where within the infarct this was taken, i.e. central or closer to the edge. Even at 24 hours there seem to be plenty of DAPI labelled cells, are these microglia? Infiltrating cells?

Response: As the reviewer suggested, we will insert a schematic indicating the peri-infarct area and infarct area in the cerebral cortex of our brain ischemia model. We have also confirmed that DAPI-labelled cells in the infarct are IBA1-positive microglia or monocytes by immunohistochemistry (shown below: Immunohistochemical staining of DAPI, NeuN and IBA1 in the infarct area 24 h after stroke onset).

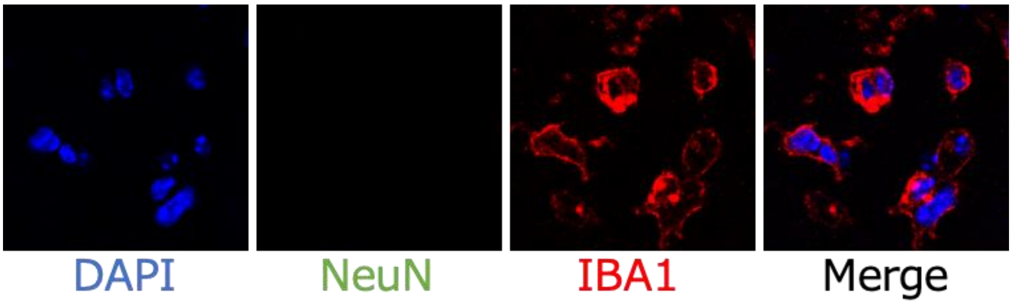

4. If the authors believe the infiltrating monocytes are to blame for some ongoing damage after stroke, and that they are activated by DJ1, there should

be fewer monocytes in the DJ1 knockout brains, have the authors quantified this? Overall I believe I saw activation status of monocytes but little evidence of changes in monocyte invasion.

Response: As the reviewer required, we are investigating the number of infiltrating monocytes in DJ-1 KO mice by immunohistochemistry. We will add the result of this experiment in our revised manuscript.

5. The clodronate experiment is neat but the authors do not comment on the changes in levels of cytokines between clodronate treated animals and controls. There is only around a 20-fold increase in TNF levels in control animals given clodronate, whereas in control animals not given clodronate the increase in TNF levels is nearly 1000-fold. Can the authors comment on this?

Response: We apologize for our mistake. This is due to the experimental artifact generated by reverse transcription and quantitative PCR in each sample. As the reviewer suggested, we are investigating whether the cytokine levels differ between clodronate treated animals and controls. We will correct this mistake in our revised manuscript.

6. The paper would be significantly boosted by evidence that DJ1 is actively released from neurons, rather than just essentially appearing because the cells are dying. This could be easily achieved by culturing neurons in ischemic conditions and measuring DJ1 levels in the supernatant over time, in correlation with cell death/apoptosis/necrosis/etc. In theory if the authors are correct and DJ1 is 'released' then there should be an elevation of DJ1 which precedes significant cell death. However, I appreciate this may be beyond the scope of the paper and is merely a suggestion to round the paper off.

Response: We agree with the reviewer's comment. We are trying to detect the extracellular release of DJ-1 in the culture supernatant of dying primary neurons.

Minor comments:
None

Reviewer #3 (Significance (Required)):

Nature and significance:
This paper provides important evidence for a novel activating factor for monocytes after ischemic stroke
Comparison to existing publications: the majority of papers focusing on DJ1 are interested in its role in Parkinson's/neurodegeneration, to find it as a novel activating factor in ischemic stroke is interesting.

Audience:
this paper will be of interest to those studying inflammation in stroke, but potentially also those interested in the role of DJ1 in neurodegeneration

My expertise:
my expertise is in the role of inflammation in ischemia
