# Supplementary material for: Extracellular DJ-1 induces sterile inflammation in the ischemic brain
Source: PLoS Biol. 2021 May 20;19(5):e3000939. doi: 10.1371/journal.pbio.3000939 (PMC8136727; doi:10.1371/journal.pbio.3000939)
Supplement: Attachment — Submitted filename: Nakamura_response to reviewers.doc [file pbio.3000939.s017.doc]

**Point-by-point reply to Reviewer #1**

**MAJOR:** 
*- The actual function of DJ-1 as a DAMP of its own is not clearly demonstrated in the present work. The in vitro part of this study uses recombinant DJ-1 (i.e. the purified peptide) in either BMDM or HEK cell cultures. However, it is very likely that in vivo DJ-1 forms complexes with other DAMP molecules (nucleic acids, lipids, histones, S100 proteins, etc.) as is known very most other DAMP classes. Therefore, interpretation of the in vitro experiments for the in vivo situation in this case is not quite possible. I suggest to add an in vitro experiment in which DJ-1 is added to BMDMs either together with brain homogenate or the 15-25 kDa fraction, at increasing doses in order to test the effect of DJ-1 in a comparable matrix as for the in vivo situation.*

Response: Thank you for this useful comment. As per your suggestion, we investigated whether the co-stimulation with brain homogenate affected the DAMP activity of a recombinant DJ-1 protein in bone marrow-derived dendritic cells (BMDCs) since BMDCs rather than BMMs were suitable for detecting DAMP activities of brain homogenate as we had previously reported (*Nature Medicine* 2012,18:911-917). As shown in **new Fig. S1**, the brain homogenate had IL-23p19-inducing activity in BMDC, whereas the costimulation with brain homogenate did not remarkably change the IL-23p19-inducing activity of a recombinant DJ-1 protein. Even if the amount of DJ-1 was changed from 0.1 to 1 μM, the addition of brain homogenate did not remarkably increase the IL-23p19-inducing activity. In these experiments, marked IL-23p19 mRNA expression in BMDCs was induced by the recombinant DJ-1 protein alone. These results demonstrated that molecules included in the brain homogenate were not essential for the DAMP activity of DJ-1.

To support our conclusion in the *in vivo* situation, we also examined the protein–protein interaction between DJ-1 and TLRs in ischemic brain tissue by proximity ligation assay (PLA). We successfully detected the interaction of DJ-1 with TLR2 or TLR4 in the infiltrating myeloid cells (**new Fig. 4G,H**), whereas these PLA signals were not observed in the ischemic brains of DJ-1 KO mice or TLR2/4 DKO mice. Thus, the DJ-1 protein interacted with TLR2 and TLR4 *in vivo*. We demonstrated that the neutralization or deficiency of DJ-1 significantly decreases the inflammatory cytokine expression in the infiltrating cells collected from the ischemic brain (**new Fig. 5A and Fig. 6A**), indicating the indispensable role of DJ-1 in triggering cerebral post-ischemic inflammation. These results revealed that DJ-1 was the key DAMP molecule in the ischemic brain.

*- The role of DJ-1 as a DAMP would require its (very) early release and broad interaction with immunocompetent cells in the ischemic brain. However, kinetics for extracellular DJ-1 expression after stroke is rather slow (Figure 4) where infiltration of innate immune cells in fact precedes DJ-1 expression.*

Response: Thank you for this helpful comment. We previously investigated the time-dependent changes of infiltrating immune cells and demonstrated that the number of infiltrating macrophages/neutrophils reaches its peak on day 3 and decreased thereafter (*Nature Medicine* 2009,15:946-950). Nonetheless, we reconfirmed the time-dependent increase of macrophage/neutrophil infiltration and found that the number of infiltrating macrophages/neutrophils increased from 12 h to 3 days after stroke onset (**new Fig. S7**). The extracellular release of DJ-1 24 h after stroke onset was more apparent than at 12 h after stroke onset, which coincided with the beginning of macrophage/neutrophil infiltration. Thus, the extracellular release of DJ-1 in the ischemic brain coincides with the infiltration of immune cells, indicating the important role of extracellularly released DJ-1 in stimulating immune cells directly.

*Moreover, extracellular DJ-1 expression seems to be confined to the ischemic core. Thereby, interaction of DJ-1 with immune cells in the peri-ischemic area -- the vulnerable area at risk after stroke and major site of secondary neuroinflammation -- is not plausible.*

Response: Thank you for this valuable comment. We apologize for the confusing explanation in the original manuscript. DJ-1 was also extracellularly released from necrotic neurons in the peri-infarct area (**new Fig. 4D: arrowhead**), since there are many neurons at risk of ischemic cell death in the peri-infarct area that expressed DJ-1 intracellularly as a response to ischemic stress, leading to the extracellular release of this DJ-1 when ischemic stress resulted in cell death. We also quantified the interaction of infiltrating myeloid cells with extracellular DJ-1 in the ischemic brain (**new Fig. 4F**), and confirmed the similar interaction of extracellular DJ-1 with infiltrating macrophages between the peri-infarct area and the infarct area. Thus, DJ-1 was considered to trigger post-ischemic inflammation not only in the infarct area but also in the peri-infarct area.

For clarity, we inserted a schematic indicating the peri-infarct area and the infarct area (**new Fig. 4C**). The extracellular release of DJ-1 and the interaction between DJ-1 and infiltrating macrophages in the peri-infarct area have been clearly described in our revised manuscript (**new Fig. 4D, F**).

*- infarct volumes for DJ-1 KO mice and littermate controls need to be reported, ideally also behavior analysis between these groups should be added*

Response: Thank you for this helpful comment. We added the results of infarct volume and neurological deficits in DJ-1 KO mice to the revised manuscript. As shown in **new Fig. 5C and Fig. 5D**, we found no significant differences in the infarct volume and neurological deficits of DJ-1 KO mice. These results can be explained by the loss of the neuroprotective (anti-oxidative) effect of intracellular DJ-1, which was demonstrated in the previous article (*PNAS* 2007,104:18748-18753) reporting that DJ-1 deficiency exacerbated ischemic neuronal damage induced by the local injection of endothelin-1, a vasoconstrictor peptide, into the brain tissue. Because a significant infiltration of immune cells is generally observed in the MCAO model, which generates a much larger infarct region than the endothelin-1 injection model, the neurotoxic effect of inflammation induced by extracellular DJ-1 in the MCAO model should be much greater compared to the local endothelin-1 injection model. Indeed, we found that the expression levels of inflammatory cytokines were significantly decreased in the infiltrating immune cells collected from DJ-1 KO mice compared with those from WT mice (**new Fig. 5A**). When we administered the anti-DJ-1 antibody to neutralize extracellular DJ-1, we observed a significant reduction in inflammatory cytokine expression and ischemic neuronal damage (**new Fig. 6**). Therefore, these results suggest that no significant difference in the infarct volume and neurological deficits of DJ-1 KO mice can be explained by the cancellation between the neurotoxic (inflammatogenic) effect of extracellular DJ-1 and the neuroprotective (anti-oxidative) effect of intracellular DJ-1. An explanation of this has been added to the *Discussion* section of the revised manuscript.

**MINOR:** 
*- the results of the behavior analysis are not convincing. It seems that both groups have similar deficits in the acute phase as well as up to 5 days after the lesion, significant difference only at 7d post-stroke. This does not fit with a function of DJ-1 as a DAMP in the acute phase driving an acute inflammatory response.*

Response: Thank you for this useful comment. We agree with this concern. We further investigated the neurological deficits of mice treated with control IgG or anti-DJ-1 antibody. We found that improved neurological deficits following the administration of anti-DJ-1 antibody were observed from day 3 to day 7 after stroke onset compared with control mice (**new Fig. 6G**). This result suggested the important role of extracellularly released DJ-1 as a DAMP in the acute phase of ischemic stroke.

*- results in Figure 4 E,F need to be quantified to show relevant interaction of innate immune cells with DJ-1 expressing cells. This should be demonstrated not only for the infarct core (which is anyways already dead tissue) but also for the perilesional tissue at risk.*

Response: Thank you for this helpful comment. We quantified the interaction of infiltrating myeloid cells with extracellular DJ-1 in the peri-infarct area and infarct area (**new Fig. 4F**) and found a similar interaction of these between the peri-infarct area and infarct area.

**Point-by-point reply to Reviewer #2**

**Major points:** 
*1. Figure 2 D: What is the response of single knockout BMMs of Tlr2 or Tlr4 on DJ-1?*

Response: Thank you for this valuable comment. We investigated the mRNA expression levels of inflammatory cytokines in *Tlr2*- or *Tlr4*-deficient BMMs stimulated by DJ-1, and found that these expression levels were partially decreased by either *Tlr2* or *Tlr4* deficiency (**new Fig. 2D**).

*2. Why did the authors skipped the ELISAs of stimulated Tlr2/4 double knockout BMMs?*

Response: Thank you for posing this important question to us. We investigated the DJ-1-induced expression of inflammatory cytokines in the culture supernatants by ELISA. We confirmed that the protein expression levels of inflammatory cytokines were partially decreased by single *Tlr2* or *Tlr4* deficiency and almost completely diminished by *Tlr2/4* double deficiency (**new Fig. 2E**). We used bone marrow–derived dendritic cells (BMDCs) for the measurement of IL-23 protein because the IL-23 protein expression in BMMs was not much enough to compare between WT and TLR-deficient BMMs.

*3. The display of the data is not satisfactory. For the sake of transparency, bar graphs should not be used. Instead, scatter dot plots like in Figure 4-6 and 95% confidence intervals, SDs or box with whiskers better illustrate the amount of independent samples. It is also not transparent what n means in the context of the displayed data (e.g. for Figure 2D). If these are independent experiments it is not enough to show technical replicates or repeated plating as independent n but rather independent experiments (isolation of BMMs from independent animals). The authors should clarify this, nested data from one animal should be avoided, or explicitly stated (and needs different statistics, too).*

Response: Thank you for calling our attention to this important point. We have changed the bar graphs to box-whisker plots (**new Fig. 1-6**). All of the results shown in **new Fig. 1-3** were obtained by triplicate measurements and confirmed by two or three independent experiments. The reproducibility of each *in vitro* experiment and the number of mice used in each experiment has been clearly described in the figure legends of our revised manuscript.

*4. If DJ-1 is released from isolated neurons it should be enriched from the medium of primary neurons subjected to oxygen-glucose deprivation and if transferred to BMMs act on the secretion of cytokines. This action might be blocked if neurons from DJ-1 knockout mice have been used or if supernatants have been incubated with anti-DJ-1 antibody.*

Response: Thank you for this valuable comment. As shown in **new Fig. S6A**, we successfully detected the extracellular release of DJ-1 in the culture supernatant of primary neurons subjected to oxygen-glucose deprivation (OGD). The culture supernatant of primary neurons subjected to OGD for 24 h induced the expression of IL-23 in BMDCs, and this IL-23-inducing activity was significantly attenuated by the addition of anti-DJ-1 antibody (**new Fig. S6B**), although the extracellularly released DJ-1 seemed to be considerably diluted by culture medium. Therefore, these results were consistent with our conclusion that the extracellular release of DJ-1 from dying neurons triggered the production of inflammatory cytokines in myeloid cells.

*5. Why did the authors conclude that the immune-stimulatory action of released DJ-1 does not stimulate resident immune cells of the brain like astrocytes or microglia?*

Response: Thank you for pointing out this important issue. To clarify this issue, we compared the expression of inflammatory cytokines in the astrocytes and microglia isolated from post-ischemic day 1 brain between control-IgG-administered and anti-DJ-1-antibody-administered mice. As shown in **new Fig. S8**, the administration of anti-DJ-1 antibody did not markedly decrease the inflammatory cytokine expression in the isolated astrocytes and microglia on day 1 after stroke onset. Since the inflammatory cytokine expression in astrocytes and microglia were relatively low in comparison with macrophage/neutrophils, the extracellular DJ-1 appeared to be important for the activation of infiltrating macrophage/neutrophils. This result is consistent with that of our previous report demonstrating with bone marrow chimeric mice that the infiltrating macrophage/neutrophils, but not residential microglia and astrocytes, were responsible for TLR2- and TLR4-mediated ischemic brain injury and inflammatory cytokine expression (*Nature Medicine* 2012,18:911-917).

*6. Figure 4 C: How do the authors exclude that DJ-1 is released in vesicles? Experiments that really explore whether it's extracellular DJ-1 are not presented. Please complement the experiments with live cell imaging of DJ-1 fusion to a fluorophore expressed in neurons derived from DJ-1 knockout animals and fluorescence fusion of either TLR2 or 4 in BMMs to show that released DJ-1 from neurons is binding to these receptors by FLIM/FRET or by proximity ligation assays.*

Response: Thank you for this valuable comment. We could detect DJ-1 protein in the brain homogenate or culture supernatant from primary neurons subjected to OGD even when extracellular vesicles were depleted by ultracentrifugation and filtration; therefore, DJ-1 was released outside the cells or vesicles. Indeed, the increase in DJ-1 protein in the culture supernatant was correlated with the neuronal cell death (decrease of β-actin in the cellular lysate) (**new Fig. S6A**). DJ-1 was likely to be passively released rather than be actively released from dead neuronal cells.

As per your suggestion, we examined the protein–protein interaction between DJ-1 and TLRs by proximity ligation assay (PLA) in the ischemic brain. As shown in **new Fig. 4G,H**, the protein–protein interaction between DJ-1 and TLR2 or between DJ-1 and TLR4 was successfully detected in the infiltrating CD11b^+^ myeloid cells whereas the PLA signals were not detected in the ischemic brain of DJ-1 KO mice or TLR2/4 DKO mice. DJ-1 was majorly released from dead neurons but not from microglia/macrophages in the ischemic brain (**new Fig. 4A and Fig. S4A**); therefore, the extracellularly released DJ-1 from ischemic neurons was bound to TLR2 and TLR4 in infiltrating myeloid cells.

*7. Figure 5: Did the authors isolate the same amount of cells from sham mice for comparison of mRNA levels? What is the difference in stroke volume in WT and DJ-1 knockout mice given the cell-autonomous action of DJ-1 in neurons etc.? Data not shown does not suffice because it is an important experiment.* *What happens if the authors compare the effects to the contralateral hemisphere of individual mice and does the enrichment strategy really underpins the statement that these are invading immune cells? Were the infarct sizes controlled to exclude a bias towards decreased stroke volumes as inflammation is a dependent variable on stroke volume? Why do the authors show relative levels to sham and not the raw data of all animals?*

Response: Thank you for raising this important point. To address it, first, we added the results of infarct volumes and neurological deficits of DJ-1 KO mice in the revised manuscript (**new Fig. 5C,D**). Although similar amounts of cells could be isolated from sham-operated WT or DJ-1 KO mice, we have replaced the result showing the relative levels to sham with the result showing the raw data of all animals (mRNA expression level of inflammatory cytokines relative to *Hprt*) (**new Fig. 5A**) as the reviewer suggested; nevertheless, the inflammatory cytokine expression levels were significantly decreased in the population enriched with infiltrating immune cells collected from the ischemic brain of DJ-1 KO mice. A similar result was obtained when we compared the effects to the contralateral hemisphere of individual mice (shown below). We also confirmed the enrichment of infiltrating immune cells from the ischemic brain by Percoll-gradient centrifugation (**new Fig. S8A**). Thus, the production of inflammatory cytokines in infiltrating immune cells was significantly decreased in DJ-1 KO mice, even if infarct sizes were similar between DJ-1 KO and WT mice and were controlled to exclude a bias arising from brain edema as described in Method section.

This phenomenon could be explained by the cancellation between the inflammatogenic (neurotoxic) effect of extracellular DJ-1 and the anti-oxidative (neuroprotective) effect of intracellular DJ-1 in the ischemic brain. A previous study demonstrated that a larger infarct was generated in DJ-1 KO mice by the injection of endothelin-1 into the brain tissue compared to WT mice (*PNAS* 2007, 104:18748-18753). In our study, we used the middle cerebral artery occlusion (MCAO) model, which induced a much larger infarct around MCA territory and severe infiltration of immune cells compared to the endothelin-1 injection model. The neuroprotective effects of intracellular DJ-1 likely predominate in endothelin-1-induced infarcts whereas, in more severe MCAO-induced infarcts, inflammatory responses are more important. Indeed, we demonstrated that the inflammatory cytokine expression and infarct volume were decreased by the administration of antibodies to neutralize extracellular DJ-1 in our MCAO model (**new Fig. 6A,B,E**), suggesting that the genetic DJ-1 deficiency impaired both the neuroprotective effect of intracellular DJ-1 and the neurotoxic inflammation triggered by extracellular DJ-1 in our MCAO model. These explanations have been added to the Discussion section of the revised manuscript.

*8. The problem of causal inference is true for the treatment with DJ-1 antibody. It is possible that DJ-1 antibody treatment results in smaller infarcts for different reasons than those that are hypothesized by the authors? Thus, inflammation is reduced and for example Il23a or Tnf are reduced as a consequence of smaller infarction. What happens, if TLR2/4 knockout mice subjected to antibody treatment or if the infarcts are titrated by timing of the occlusion period to the same size? Is Anti-DJ-1 antibody still decreasing Tnf on the mRNA level? Of note, clodronate experiments should be visualized to allow readers to understand how successful macrophages vanished from the brain.*

Response: Thank you for calling our attention to this important point. We investigated the inflammatory cytokine expression in *Tlr2/4*-deficient mice that were treated with antibodies. As previously reported (*Nature Medicine* 2012,18:911-917) and shown in **new Fig. 6C**, *Tlr2/4* deficiency significantly decreased the inflammatory cytokine expression in the immune cell–enriched population from the ischemic brain, copared to WT mice. However, the administration of anti-DJ-1 antibody did not change the inflammatory cytokine expression in *Tlr2/4*-deficient mice. These results support our conclusion that extracellular DJ-1 activates TLR2 and TLR4 in infiltrating myeloid cells to trigger the cerebral post-ischemic inflammation. We also confirmed the successful depletion of macrophages by the administration of chlodronate (**new Fig. S9A**).

*9. The authors state that inflammation rather than extracellular DJ-1 triggers neuronal cell death (Fig S6). First, Neuro2A cannot be considered a neuron and it is extensively demonstrated that neurons are distinct to immortalized cells and rapidly lose their phenotype. This experiment would be more meaningful if primary neurons would have been investigated and studied over a longer period than 24h, which is too short to exclude that DJ-1 induces apoptosis over several days.*

Response: Thank you for this helpful comment. By using primary neurons, we successfully confirmed the neurotoxicity of the culture supernatant from DJ-1 stimulated BMMs (**new Fig. S10A**). DJ-1 did not directly induce neuronal cell death in primary neurons, even when primary neurons were cultured with DJ-1 for 72 h (**new Fig. S10B**). Thus, DJ-1 activated myeloid cells to trigger the inflammation that induced neuronal cell death.

*10. Figure S7: What is the effect of clodronate on the immune cells and what is the effect of combined treatment on the stroke volume?*

Response: Thank you for pointing this out. The administration of clodronate significantly decreased the infiltration of macrophages in the ischemic brain (**new Fig. S9A**). Further administration of anti-DJ-1 antibody to clodronate-treated mice did not change the infarct volume or neurological deficits compared to control IgG- and clodronate-administered mice (**new Fig. S9C**). These results demonstrated that ischemic neuronal injury was promoted by the extracellular DJ-1-induced activation of macrophages.

*11. Table S2: The authors show the mortality without statistics and percentage although group sizes were not equal in the beginning. A statistical analysis is not shown although the data were interpreted. Please change this. Reporting should be in line with the ARRIVE guidelines and mortality, exclusion and inclusion criteria, time and reason of death etc. should be reported for each in vivo experiment.*

Response: Thank you for this useful comment. This information was added in **new Table S1A,B**. The survival rates of all *in vivo* experiments were statistically analyzed, and the exclusion/inclusion criteria has been described in the Method section of our revised manuscript.

*12. A Score should be displayed as median and not as mean since these are not metric data. Post-hoc correction for multiple testing should be performed. The authors overstretch this functional readout a lot. It is not described whether blinding or randomization was performed and easy to apply behavioural tests were not used. The reviewer understands that this might be not the scope of this publication but one cannot draw the conclusion that there is functional improvement by the treatment.*

Response: Thank you for this helpful comment. The neurological scores were displayed as median and statistically analyzed by multiple comparison test with *post hoc* correction (**new Fig. 6G**). Blinding and randomization were performed for the evaluation of neurological deficits, which has been clearly described in the Method section of our revised manuscript.

*13. Did the authors investigate whether DJ-1 antibody is not resulting in a western blot immunoreactivity in brain tissue derived from knockout mice? Did they test immunohistochemistry with their DJ-1 antibody?*

Response: Thank you for these insightful questions. We confirmed the specificity of the DJ-1 antibody by Western blotting analysis and immunohistochemistry using DJ-1-deficient tissue (**new Fig. S4B,C**).

***Minor points:** 

Display of graphs as scatter dot plots and SD or 95% CI would be preferred. 
BMM - please spell out at first usage on page 4 in the heading 
PGN please spell out at first usage (TLR2 ligand)* 

Response: Thank you for these useful comments. We have corrected these errors in the revised manuscript.

**Point-by-point reply to Reviewer #3**

***Major comments:** 

1. Details of the percoll gradient for immune cells in the brain are not clear. How are the authors guaranteeing they have infiltrating monocytes vs microglia/astrocytes/etc.*

Response: Thank you for calling our attention to this important point. We confirmed the enrichment of infiltrating microglia, macrophages, and neutrophils by Percoll-gradient centrifugation (**new Fig.S8A**), which was described in our previous article (*Nature Medicine* 2009,15:946-950). Although this immune cell–enriched population from the ischemic brain also included brain cells such as astrocytes, we also confirmed that the major source of inflammatory cytokine was infiltrating macrophage/neutrophils rather than microglia or astrocytes (**new Fig.S8C**). In our revised manuscript, we replaced “infiltrating immune cells” with “the population enriched with infiltrating immune cells collected from ischemic brain”.

*2. There is a comment in the discussion saying that data is not shown on infarct volumes in DJ1 knockout mice, I believe these would considerably add to the manuscript and I believe they should be included.*

Response: We appreciate this helpful comment. We added the results on infarct volume and neurological deficits of DJ-1 KO mice to our revised manuscript (**new Fig. 5C,D**). DJ-1-deficiency did not change the infarct volume and neurological deficits in comparison with WT mice, even if the expression of inflammatory cytokines was significantly decreased in the immune cell–enriched population collected from DJ-1-deficient ischemic brain (**new Fig.5A**). We considered this to be a result of the cancellation between the neurotoxic inflammation triggered by extracellular DJ-1 and the anti-oxidative effect (neuroprotection) by intracellular DJ-1. It was reported by Aleyasin *et al*. that a larger infarct is generated in DJ-1 KO mice than WT mice by using the endothelin injection model of focal ischemia (*PNAS* 2007,104:18748-18753). In our transient middle cerebral artery occlusion (MCAO) model, a much larger infarct and severe infiltration of immune cells occurred compared to the endothelin injection model. Therefore, the role of inflammation should be much more important, given that the neutralization of extracellular DJ-1 by antibody administration decreased the infarct volume and the inflammatory cytokine expression in the immune cell–enriched population collected from the ischemic brain (**new Fig. 6A,E**). DJ-1 deficiency impaired both the neuroprotective effect of intracellular DJ-1 and the inflammatogenic effect of extracellular DJ-1, suggesting the importance of neutralizing extracellular DJ-1 in the treatment of ischemic stroke. These explanations have been added in the Discussion section of the revised manuscript.

*3. The immunohistochemistry for DJ1 is supposed to be 'infarct', it would be useful to have a schematic to demonstrate where within the infarct this was taken, i.e. central or closer to the edge. Even at 24 hours there seem to be plenty of DAPI labelled cells, are these microglia? Infiltrating cells?*

Response: Thank you for calling our attention to this important point. We inserted a schematic indicating the peri-infarct area and infarct area in the cerebral cortex of our brain ischemia model (**new Fig. 4C**). We also confirmed that DAPI-labelled cells in the infarct are IBA1-positive microglia or macrophages by immunohistochemistry (shown below: immunohistochemical staining of DAPI, NeuN, and IBA1 in the infarct area 24 h after stroke onset).

*4. If the authors believe the infiltrating monocytes are to blame for some ongoing damage after stroke, and that they are activated by DJ1, there should be fewer monocytes in the DJ1 knockout brains, have the authors quantified this? Overall I believe I saw activation status of monocytes but little evidence of changes in monocyte invasion.*

Response: We appreciate this helpful comment. We compared the number of infiltrating myeloid cells in the ischemic brain between WT and DJ-1 KO mice by immunohistochemistry and found no significant difference (**new Fig. 5B**). This result is consistent with that of the anti-DJ-1 antibody treatment experiment showing that the number of infiltrating myeloid cells was not significantly altered by the administration of antibodies (**new Fig. 6D**). These results led us to conclude that the deficiency or neutralization of extracellular DJ-1 did not significantly inhibit the infiltration but suppressed the inflammatory cytokine production in infiltrating immune cells, revealing the important role of DJ-1 in triggering the inflammatory cytokine production in the ischemic brain.

*5. The clodronate experiment is neat but the authors do not comment on the changes in levels of cytokines between clodronate treated animals and controls. There is only around a 20-fold increase in TNF levels in control animals given clodronate, whereas in control animals not given clodronate the increase in TNF levels is nearly 1000-fold. Can the authors comment on this?*

Response: Thank you for pointing this out. We apologize for our mistake. This was due to the experimental artifact generated by reverse transcription and quantitative PCR in each sample. We re-evaluated the results of quantitative PCR and corrected the values for inflammatory cytokine expression in the revised manuscript (**new Fig. 6B**).

*6. The paper would be significantly boosted by evidence that DJ1 is actively released from neurons, rather than just essentially appearing because the cells are dying. This could be easily achieved by culturing neurons in ischemic conditions and measuring DJ1 levels in the supernatant over time, in correlation with cell death/apoptosis/necrosis/etc. In theory if the authors are correct and DJ1 is 'released' then there should be an elevation of DJ1 which precedes significant cell death. However, I appreciate this may be beyond the scope of the paper and is merely a suggestion to round the paper off.*

Response: Thank you for calling our attention to this important point. In the original version of the manuscript, we used the term “release” in the sense of passive release by cellular membrane destruction due to severe cellular damage or cell death rather than active release (secretion) by dying cells. We evaluated the time course of DJ-1 release into the culture supernatant from the primary neuron subjected to oxygen-glucose deprivation, and found that the increase in DJ-1 protein in the culture supernatant was correlated with the neuronal cell death (decrease of β-actin in the cellular lysate) (**new Fig. S6A**). Therefore, DJ-1 was likely to be passively released from dead neuronal cells. To avoid this confusion between passive release and active release, we clearly state “passive release from dead neuronal cells” to describe the extracellular release of DJ-1 in the revised manuscript.
