# Supplementary material for: Extracellular DJ-1 induces sterile inflammation in the ischemic brain
Source: PLoS Biol. 2021 May 20;19(5):e3000939. doi: 10.1371/journal.pbio.3000939 (PMC8136727; doi:10.1371/journal.pbio.3000939)
Supplement: Attachment — Submitted filename: Nakamura_response to reviewers#2.docx [file pbio.3000939.s018.docx]

**Point-by-point reply to Reviewer #1**

*Reviewer #1: The authors have extensively and comprehensively addressed all concerns and the manuscript is much improved. After having reviewing comments ignored by a number of authors it is refreshing to see their attitude was conversational, rather than confrontational and suggested changes were discussed in light of the literature and their own new data. Thank you for making reviewing more enjoyable than it has been recently!*

Response: Thank you very much for your comments on our manuscript.

**Point-by-point reply to Reviewer #2**

*Reviewer #2: The authors did an excellent job in addressing all of my concerns. I have no additional comments*

Response: We highly appreciate the reviewer for reviewing our manuscript

**Point-by-point reply to Reviewer #3**

*Reviewer #3: The manuscript has substantially improved by the additional experiments and the transparency of the way the data are displayed. All of my concerns have vanished and I congratulate the authors to this novel and important contribution towards understanding the pathophysiology of stroke.*

Response: Thank you for reviewing our manuscript and for providing supportive comments.
