# Supplementary material for: Extracellular DJ-1 induces sterile inflammation in the ischemic brain
Source: PLoS Biol. 2021 May 20;19(5):e3000939. doi: 10.1371/journal.pbio.3000939 (PMC8136727; doi:10.1371/journal.pbio.3000939)
Supplement: Attachment — Submitted filename: Nakamura_response to editor#3.docx [file pbio.3000939.s019.docx]

**Point-by-point reply to Editor**

*1) Ethics Policy Request - Thank you for including an ethics statement in your manuscript. The ethics statement is currently missing information regarding the specific national or international regulations/guidelines to which your protocol adheres. Would you please add a sentence to your ethics statement with this information? For example, you could add the sentence saying “Experiments adhered to __ guidelines." (Fill the blank with relevant national or international guidelines). Please note that the institutional or accreditation organization guidelines (Such as AALAC) do not meet this requirement.*

Response: We added the scentence of *“The animal care and use protocol adhere to the ARRIVE guidelines (“Animal Research: Reporting of In Vivo Experiments”; https://arriveguidelines.org/)”* on our manuscript.

*2) Data Policy Request - Thank you for providing the numerical values used in your figures as S1_data. Would you please add a sentence to each figure legend that that refers to this supplemental file? For example, you could add a sentence to the end of each figure legends saying “The data underlying this figure can be found in S1_data”. Please make sure to include this sentence in the supplementary figure legends as well.*

Response: We added the scentence of *“The data underlying this figure can be found in S1_data”* on our each figure legends and supplementary figure legends. *3) Please also make sure that the S1_data file contains a legend describing what it is.*

Response: We added the legends on S1_data file.
